# Peer-led small groups: Are we on the right track?

**DOI:** 10.1007/s40037-017-0370-0

**Published:** 2017-08-18

**Authors:** Fraser Moore

**Affiliations:** McGill University Centre for Medical Education, Montreal, Quebec Canada

**Keywords:** Small group teaching, Peer teaching

## Abstract

**Introduction:**

Peer tutor-led small group sessions are a valuable learning strategy but students may lack confidence in the absence of a content expert. This study examined whether faculty reinforcement of peer tutor-led small group content was beneficial.

**Methods:**

Two peer tutor-led small group sessions were compared with one faculty-led small group session using questionnaires sent to student participants and interviews with the peer tutors. One peer tutor-led session was followed by a lecture with revision of the small group content; after the second, students submitted a group report which was corrected and returned to them with comments.

**Results:**

Student participants and peer tutors identified increased discussion and opportunity for personal reflection as major benefits of the peer tutor-led small group sessions, but students did express uncertainty about gaps in their learning following these sessions. Both methods of subsequent faculty reinforcement were perceived as valuable by student participants and peer tutors. Knowing in advance that the group report would be corrected reduced discussion in some groups, potentially negating one of the major benefits of the peer tutor-led sessions.

**Discussion:**

Faculty reinforcement of peer-tutor led small group content benefits students but close attention should be paid to the method of reinforcement.

## What this paper adds

Peer tutor-led small group sessions can increase student discussion but students may lack confidence in their learning in the absence of a faculty content expert. The need for strategies to overcome this have not been studied in medical school. This study shows that some students do express uncertainty following peer tutor-led small group sessions. Two different methods of reinforcement not only helped some students but also increased student reflection. However, student awareness in advance that reinforcement would occur reduced discussion in some groups and risks negating a major benefit of peer tutor-led small groups.

## Introduction

Small group teaching is widely used in undergraduate medical education. Traditional small group teaching involves faculty-led small groups. Student-led small groups are led by students at the same level of training as the learners (peers) or students at more advanced levels (near-peers) [1–4]. Student-led small groups are feasible and may be as effective as faculty-led small groups [5–7]. This has been explained by theories of cognitive congruence (peers and near-peers think about and approach problems in a similar manner) and social congruence (peers and near-peers relate to each other more easily leading to a better learning environment) [8–11].

There are multiple other potential advantages to a peer tutor-led small group including providing students with teaching experience [1, 4]. However, only a few studies have examined peer-led small group teaching in medical school [8, 12–16]. Even fewer studies have addressed whether students have confidence in their own learning experience with a peer tutor; students might perceive the absence of a content expert as a negative if they felt they did not receive sufficient confirmation that their ideas and answers were correct [10, 12, 15, 17]. A possible solution would be subsequent reinforcement of the small group content by a faculty expert, but this has not been studied.

The goal of this study was to determine if we are ‘on the right track’ with peer tutor-led small groups; do students feel unsure or uncomfortable about their grasp of the material after a peer tutor-led small group and, if so, can faculty reinforcement of the small group content help? Questionnaires completed by student participants after peer-led small groups and faculty-led small groups were compared and the peer tutors were interviewed. Two methods of faculty reinforcement of the peer tutor-led small group material were compared and both student participants and peer tutors were asked if this was useful or necessary.

## Methods

The study was conducted during the McGill Medical School ‘Human Behavior’ course lasting 8 weeks in October–November of year 2 of the 4‑year medical school curriculum. Teaching occurs in lectures, the anatomy laboratory, and a total of 17 small group teaching sessions. Ten small groups each consist of 12–15 students. The same groups of students had worked together in previous faculty-led small group sessions during medical school.

### Small group sessions

Three of the 17 small group sessions were analyzed. One session was faculty-led and two sessions were peer-led. They occurred in October and November 2014. A different type of reinforcement was examined after each of the peer-led small group sessions (as explained below). A greater number of sessions were not analyzed because it was important that students were able to recall each individual session referred to in the questionnaires (see below), the format of each of the sessions (peer or faculty-led), and the method of reinforcement used.

Each session was 2 h in length and consisted of a clinical case followed by a series of questions covering anatomical localization, diagnosis, social aspects such as loss of autonomy, and ethical aspects such as withdrawal of care. Students received the case and questions in advance but no preparation was required other than reading the case. Small group session 2 (SG2) was led by a peer tutor and covered the use of the neurological examination to localize a lesion of the spinal cord. Reinforcement occurred 6 days later with a faculty lecture on the spinal cord that included review of the small group material. Small group session 12 (SG12) was led by a peer tutor and covered head trauma and cerebral herniation. Reinforcement occurred by requiring each group to submit a written report with their group responses to each question. This was returned to them with comments. Small group session 15 (SG15) was led by a faculty tutor and covered a case of coma and brain death. Students participating in the peer tutor-led SG2 and SG12 only received marks for attendance and were not evaluated by the peer tutors. Students in SG15 (and other faculty-led small groups in the course) were evaluated on both attendance and participation.

### Selection and preparation of tutors

Peer tutors were randomly selected from a list of members of the 10 existing small groups. This was done by an administrator from the faculty of medicine who was not involved in either this study or teaching. Selected students were sent an explanation of the study and invited to participate. The 10 peer tutors met as a group in person with the author for 30 min, 2 days prior to SG2. The study was explained and each peer tutor signed an informed consent form. Peer tutors were expected to guide group discussion, involve their colleagues, and ensure that students treated the sessions seriously. No further instruction was provided regarding how to be an effective tutor or lead a small group. Peer tutors also did not receive any extra instruction regarding the content of the small groups. The ten peer tutors each acted as the peer tutor for SG2 *and* SG12.

### Outcome measures

The first outcome measure was an on-line questionnaire administered after each of the small group sessions (SG2, SG12, SG15). The same questionnaire was used following each of the three sessions. It assessed student opinion about the functioning of the small group and the value of the session for their learning (Table 1). The second outcome measure consisted of semi-structured interviews conducted in-person by the author with each of the peer tutors. These interviews occurred between December 2014, and May 2015. They assessed peer tutor opinion about the function of the small group and their experience as a peer tutor (was it difficult, stressful, beneficial, etc …). The third outcome measure consisted of two online questionnaires completed by all students except the peer tutors concerning the two methods of reinforcement; the review lecture following SG2 and the report following SG12. These questionnaires assessed student participant opinion about whether these reinforcement measures were helpful and necessary (Table 2).

**Table 1:** Participant feedback regarding the small group experience. Each question was answered on a 5-point Likert scale from 1=strongly disagree to 5=strongly agree. Results are expressed as the average of all student responses for a given question. (*n* refers to the number of questionnaires received, not the number of students attending the small groups)

Item	SG2 (peer tutor) *n* = 82	SG12 (peer tutor) *n* = 57	SG15 (non-peer) *n* = 36
1. I enjoyed this small group session	4.25	3.93	4.28
2. This small group session helped me to learn the material presented	4.18	3.97	4.11
3. I feel that we fully covered the required topic during this small group session	4.16	4.07	4.17
4. I feel that my group ‘cut corners’ and did not spend the time necessary to discuss the topic	1.58*	2.12*	1.94*
5. There was good discussion among group members during the small group session	4.51**	3.90**	4.31**
6. It was easy for me to express my opinion	4.35***	3.98***	4.11***
7. One or a few students dominated the discussion during the group	2.72	2.89	2.71
8. This small group session should be repeated in the course next year	4.01	3.72	4.06

*p = 0.0003 **p < 0.0001 ***p = 0.047

**Table 2:** Participant feedback regarding the follow-up lecture to small group 2 (peer tutor) and the written report for small group 12 (peer tutor). Each question was answered on a 5-point Likert scale from 1 =strongly disagree to 5 =strongly agree. Results are expressed as the mean of all student responses for a given question

*Lecture*	*(n = 57)*
It was helpful to review the small group material during the lecture	4.55
The lecture improved my understanding of the small group material	4.27
Reviewing the small group material in the lecture was not helpful for me	1.54
I would have preferred to use the time during the lecture to cover other material, rather than reviewing the small group	1.81
*Written report*	*(n = 60)*
It was helpful to submit the written report following the small group	3.72
The feedback our group received regarding the written report improved my understanding of the material discussed in the small group	3.98
Submitting the written report was unnecessary	2.57
Small group time would have been more appropriately used for group discussion, rather than preparing the report	2.60

### Data analysis

Mean responses to questions using Likert scales were compared using one-way analysis of variance (standard weighted-means analysis, F statistic) (Table 1). Responses to questions regarding the two reinforcement methods were compared with descriptive statistics alone because individual questions were not identical (Table 2). Qualitative content analysis of student comments from the questionnaires and of the peer tutor interviews was used to organize responses into themes [18]. Approval for the study was obtained from the Institutional Review Board of McGill University.

## Results

### Small group learning experience – student feedback

Average student responses to questions are presented in Table 1. Results for only three of the eight questions differed significantly when comparing the three small group sessions. The results for the faculty (non-peer) led small group (SG15) were intermediate between those for the two peer tutor-led small groups (SG2 and SG12). Student written comments emphasized that the peer tutor-led small groups increased student participation:

*It was definitely easier for me to ask questions amongst my peers – I was more comfortable asking for clarification on something we already covered where I may have been too embarrassed with a tutor. More people participated and shared their knowledge. Students were less hesitant to draw on the board as they weren’t afraid to make an error*.

Other students emphasized the benefits of cognitive congruence: *‘Students understand how we learn, and therefore can explain material in a very simple and clear way’. *Negative comments focused almost exclusively on the uncertainty of not knowing if the answers discussed in the sessions were correct: *‘The only main frustration was that we didn’t know if our reasoning was correct or not’ *and* ‘I would have preferred to have a (faculty) group leader …to ensure that we were on track’.*

### Small group learning experience – peer tutor perception

Multiple peer tutors felt that the peer tutor-led small group format encouraged group discussion: *‘(It was) fun and different …less formal, less intimidating …(it is) rare that (the discussion) is really a group collective (like this was).’ *Lack of expertise was identified as a negative by some peer tutors: *‘I felt almost guilty if I didn’t know enough’*. Others felt that acting as a peer tutor *‘changed how I thought about the small groups’ *and helped them realize the limitations of a content expert by understanding *‘the tutor role more and the art of medicine, the fact that answers are not always black and white.’* Many peer tutors felt they had to *‘… become more comfortable talking to people, managing people, and seeing the big picture.’ *More significantly some felt that the experience allowed them *‘increased opportunity for (personal) reflection …can I guide and help others understand?’*

### Reinforcement measures – student feedback

Student responses were generally more positive concerning the lecture than the written report (Table 2). Written comments indicated that reinforcement of SG2 in the subsequent lecture could be very beneficial for some students:

*I realized how much I had internalized when (we) went over it in lecture and I remembered everything (we) talked about.*

*The solution … presented in lecture differed slightly from the one that emerged from our group’s discussion. Seeing the slight gaps between both helped me to correct and consolidate my knowledge, as well as realize the potential for uncertainty and gray areas in medicine, particularly in how one defines the clinical problem.*

Some students commented that making the answers to the small group questions available (for example, online) would have been sufficient and therefore covering the material in lecture was unnecessary.

Preparation of the report intended to reinforce the content of SG12 helped some students because there were ‘*some topics where our explanation to each other may not have been satisfying and we would like the input of a tutor or a professor. The written feedback helps with that.’ *However, more students commented about the effect on the dynamics of the small group:

*The group focused on preparing the report rather than discussing the important points. The focus was less about everybody understanding, but rather on having a report that was perfectly done.. there wasn’t much synthesis of the knowledge …(we) never went on to explain other interesting things that were brought up during the small group.*

In other groups the report had the opposite effect and ‘*encouraged further clarification and communication between the group on each point in order to be able to clearly report the concept.’*

### Reinforcement measures – peer tutor perception

Peer tutor impressions of the two reinforcement methods were also mixed. They felt the advantage of the written report was that students knew they would be getting comments *‘on the answers we gave’*. The disadvantage in some groups was that students ‘*(tried) to encompass too much to ensure that the answer is there …it closed discussion*’. When there was no report to complete *‘people don’t hold back’ *with discussion*. *Comments about the lecture were that it ‘*was helpful to reinforce and reassure’, *but might be better if it occurred sooner after the small group (rather than 6 days later as in this case).

## Discussion

Peer tutor-led small groups can function effectively in medical school. In this study both student participants and peer tutors noted that they increased student participation. The uncertainty of not knowing ‘the right answer’ in the absence of a faculty tutor as ‘expert’ was a source of discomfort for some students as has previously been shown [12, 15]. It was also a source of anxiety for some of the peer tutors themselves. To the author’s knowledge, the use of subsequent reinforcement as a strategy to overcome this uncertainty and anxiety has not been previously reported. Both reinforcement strategies used here helped some students. Beyond reducing uncertainty these strategies may have the additional benefit of promoting student reflection over time, as some students compared ideas taken away from the small group session to those in the subsequent lecture or corrected report, helping them to realize that there can be ‘uncertainty and grey areas’ in medicine. Reinforcement may thus act as a form of distributed practice or learning [19, 20].

Knowing in advance that reinforcement will occur can potentially have a negative impact on the functioning of small groups. Although in some groups the structure imposed by the written report was positive and helped focus the group, in other groups students concentrated too much on producing the report rather than discussing the content. It may be ideal if students are not aware in advance that reinforcement will occur, as was the case here with the lecture, or if they are aware then the importance of discussion within the small group should be emphasized. This could be accomplished with the use of more open questions, framed to promote discussion. For example, students could be asked to ‘provide some of your groups ideas’, rather than specific factual answers or solutions to problems.

This study identified another benefit of peer tutor-led small group sessions. In addition to promoting student reflection on knowledge and uncertainty in medicine, peer tutors identified an opportunity for increased reflection on the role of a student as an educator. Such changes in attitudes as a result of peer teaching have been described in other programs [8] but not in medical school [7], although some authors have anticipated this benefit [3, 9]. Determining if these benefits are maintained long-term could be a direction for future study. It would also be interesting to know if peer tutors shared this insight/experience with their fellow students.

One limitation of this study is that only two peer tutor-led small group sessions were evaluated. It is possible that a greater number of sessions would enhance the functioning of peer tutor-led small groups and lead to less perceived benefit or need for faculty reinforcement [10]. A second limitation is that students were not evaluated on participation during the peer tutor-led small groups but were evaluated during the comparison faculty-led small group. If students had approached faculty-led small groups more seriously (because they were evaluated) then they may have perceived greater need for faculty reinforcement after the peer tutor-led small group. Alternatively, students may have been more relaxed when they knew they were not being evaluated during the peer tutor-led sessions, further contributing to increased discussion and learning and reducing the perceived need for faculty reinforcement. Future studies should address the impact of evaluation on peer tutor-led small group sessions. A final limitation is the possibility that the results are specific to small groups in this medical school and are not generalizable. Students with greater small group experience in medical school might not need a peer tutor-led small group to increase discussion, while students with less experience might lack the confidence or ability to take advantage of this format. The results do show the potential of both the peer tutor-led small group format and of subsequent faculty reinforcement to increase student reflection and distributed learning; readers could adapt both to the curriculum of their medical school to keep peer tutor-led small groups ‘on the right track’.
